# A case of panuveitis with hypopyon due to presumed ocular leishmaniasis in a HIV patient

**DOI:** 10.1186/s12348-014-0021-0

**Published:** 2014-08-29

**Authors:** Simon Couture, Rupesh Agrawal, Kate Woods, Diana Lockwood, Carlos E Pavesio, Peter K F Addison

**Affiliations:** Medical Retina Service, Moorfields Eye Hospital NHS Foundation Trust, London, EC1V 2PD UK; Département d’Ophtalmologie et ORL–Chirurgie cervico-faciale, Université Laval, Québec, G1TW4 Canada; National Healthcare Group Eye Institute, Tan Tock Seng Hospital, Singapore, 308433 Singapore; London School of Hygiene & Tropical Medicine, London, UK

**Keywords:** Ocular leishmaniasis, Granulomatous panuveitis, Immune reconstitution uveitis, HIV

## Abstract

**Background:**

Post-kala-azar dermal leishmaniasis is a well-known immunologic cutaneous reaction. There are few case reports of ocular leishmaniasis. It is a sight-threatening condition that needs to be rapidly recognized and treated to avoid permanent visual loss. Ocular leishmaniasis panuveitis can present with severe inflammation in patients with highly active anti-retroviral therapy (HAART)-induced immune reconstitution syndrome.

**Findings:**

A case of a 40-year-old man, human immunodeficiency virus (HIV) positive on HAART, with a presumed diagnosis of ocular leishmaniasis, is presented. He had a past history of visceral leishmaniasis and was referred to the uveitis service with rapidly worsening panuveitis and counting fingers vision in both eyes. On empirical anti-leishmania therapy and systemic steroids, the visual acuity of the left eye improved to 6/9 but remained poor in the right eye. Based on the medical history, improvement with therapy and the exclusion of other common infections, a presumed diagnosis of ocular leishmaniasis-related panuveitis was made.

**Conclusions:**

A major immune reaction against lingering parasites may play a key role in the pathogenesis of this sight-threatening and rapidly progressive condition. Both the infection and the immune reaction should be treated.

**Electronic supplementary material:**

The online version of this article (doi:10.1186/s12348-014-0021-0) contains supplementary material, which is available to authorized users.

## Findings

### Introduction

Leishmaniasis is caused by Leishmania parasites transmitted by sandflies to mammalian hosts. It is most commonly found in rural impoverished areas of the Far and Middle East; Central, Western and Eastern Europe; Africa; Asia; and Central and South America [1]. There are about two million new cases per year [1]. Three clinical syndromes have been described: the cutaneous disease, the mucocutaneaous disease and the visceral disease, also named kala-azar [2]. The cutaneous form is the commonest form. Visceral leishmaniasis (VL) is the most severe and may have a 75% to 95% mortality rate within the first 2 years if left untreated.

Post-kala-azar dermal leishmaniasis is an immunologic and cutaneous reaction in patients treated for visceral leishmaniasis [3]. In human immunodeficiency virus (HIV)-positive patients, the development of this cutaneous condition has been reported as a manifestation of immune reconstitution syndrome [4].

Ocular leishmaniasis has rarely been reported in the literature [5]-[8]. The pathogenesis of ocular leishmaniasis remains unclear. It is a sight-threatening condition, which needs to be rapidly recognized and treated to prevent permanent visual loss. Ocular leishmaniasis panuveitis has been postulated to be a part of highly active anti-retroviral therapy (HAART)-induced immune reconstitution syndrome in a co-infected HIV patient [8].

### Case report

Written informed consent was obtained from the patient for the publication of this report and any accompanying images. Our patient was originally from Eritrea. He immigrated to the UK in 2005. He had been found to be HIV-1 positive in 2008 when he presented with VL. He then had good viral load suppression with anti-retroviral therapy, but his maximum CD4 count was only 120 cells/mm^3^ (baseline 55 cells/mm^3^ at diagnosis); he had not suffered opportunistic infections. However, his VL recurred in June 2012 with a relapsing course requiring five admissions despite appropriate prophylaxis with miltefosine and/or pentamidine. He had a classical presentation of VL with fever, fatigue, anorexia, weight loss, skin rash and splenomegaly. Amastigotes of *Leishmania donovani* were found on biopsy of split skin smear, and he responded to prolonged treatment with liposomal amphotericin B.

**Figure 1:**
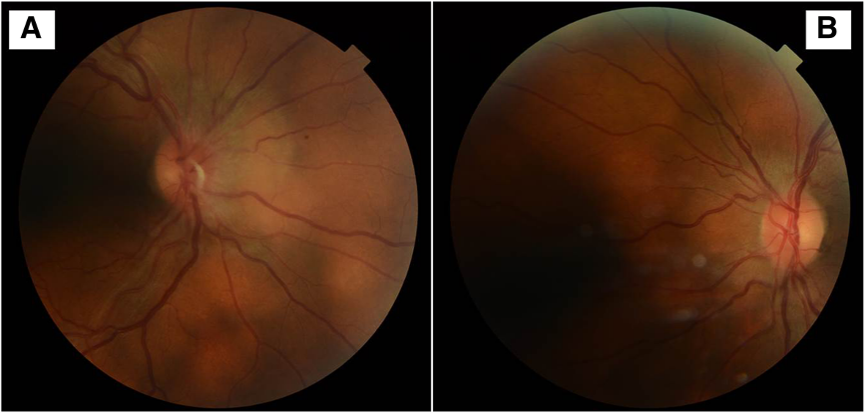
**Colour fundus photos of the disc.** Showing presence of disc oedema in the right **(A)** and left **(B)** eyes.

**Figure 2:**
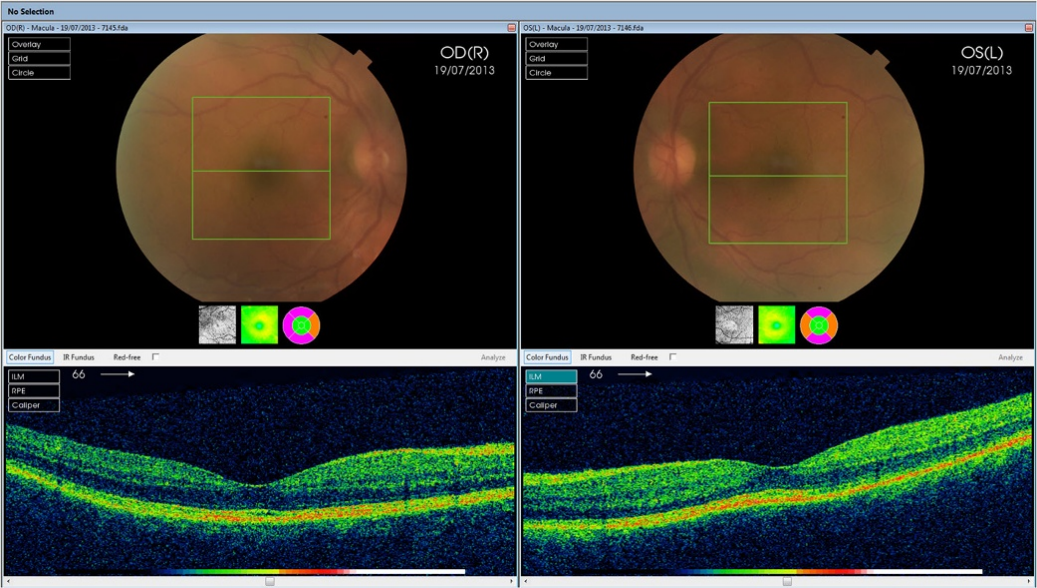
**3D-OCT scan.** Showing presence of normal macula and no evidence of fluid intraretinally or presence of any area of serous retinal detachment.

In April 2013, he presented with a recurrence of maculo-papular rash, gradual onset blurred vision, fatigue and headache less than a week after completing a prolonged course of liposomal amphotericin B for a relapse of leishmaniasis in February 2013. He was noted to have optic disc oedema in both eyes (Figure 1), and hence, neuroimaging and lumbar puncture were performed. No abnormality was seen on magnetic resonance imaging (MRI) scanning. *L. donovani* (LD) was detected by polymerase chain reaction (PCR) on cerebrospinal fluid analysis. Leishmania amastigotes were seen in (Figure 2) split skin smear taken from his rash. He was treated with 28 days of intravenous sodium stibogluconate for disseminated leishmaniasis with central nervous system involvement. There was complete clinical recovery. His CD4 count also reconstituted for the first time since diagnosis to 440 cells/mm^3^, without any change in his anti-retroviral regimen.

**Figure 3:**
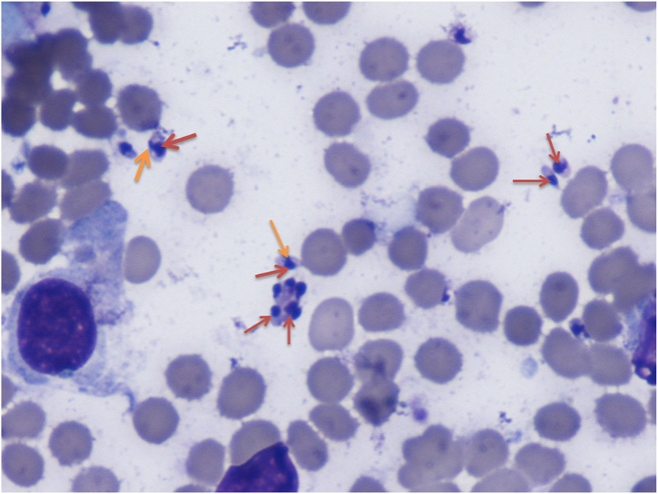
**Giemsa stain of split skin smear.** Showing presence of leishmania amastigotes (red arrows) and kinetoplast (orange arrows) on light microscopy.

In addition to anti-retroviral therapy, he was kept on oral mitefosine, as leishmania prophylaxis. He remained well until July 2013 when he re-presented with severe headache and visual impairment. His visual acuity was 6/9 in both eyes. During this episode, acute bilateral granulomatous anterior uveitis was noted with normal posterior segment examination and normal optical coherence tomography (OCT) (Figure 3). He was treated with topical steroids. Unfortunately, within days, visual acuities fell to counting fingers in both eyes. On examination, he had severe bilateral granulomatous anterior uveitis with hypopyon in the right eye, mutton fat keratic precipitates, posterior synechiae and no view of the posterior segment from either eye. He was started on hourly topical steroids along with oral prednisone 60 mg once a day. B-scan ultrasound examination in the right eye showed a temporal supra-choroidal shallow elevation of 1.2 mm with mild vitritis. All blood and radiological investigations were normal or negative including *Treponema* serology and QuantiFERON®-TB Gold (QFT-G, Cellestis Limited, Carnegie, Victoria, Australia) for detecting TB-specific T cells which were negative.

**Figure 4:**
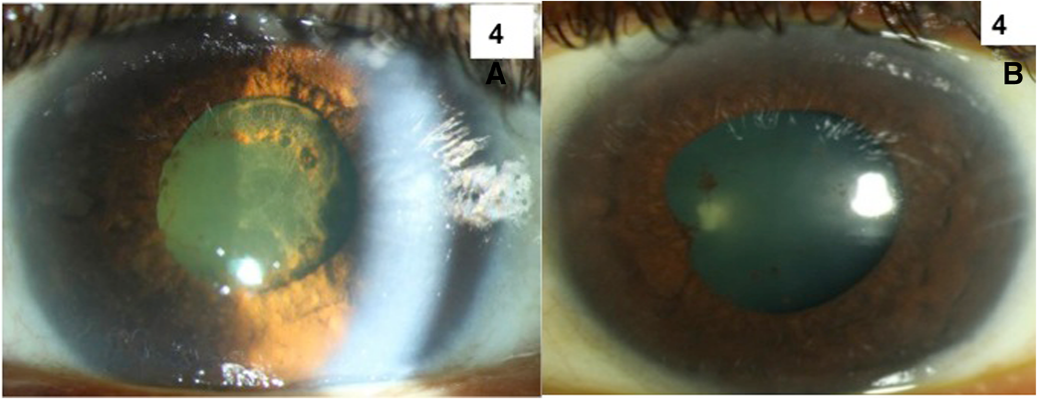
**Slit Lamp Photograph.** Slit-lamp photograph of the left eye showing presence of chronic inflammation and Descemet membrane folds **(A)** and resolution of inflammation and Descemet membrane folds after treatment **(B).**

**Figure 5:**
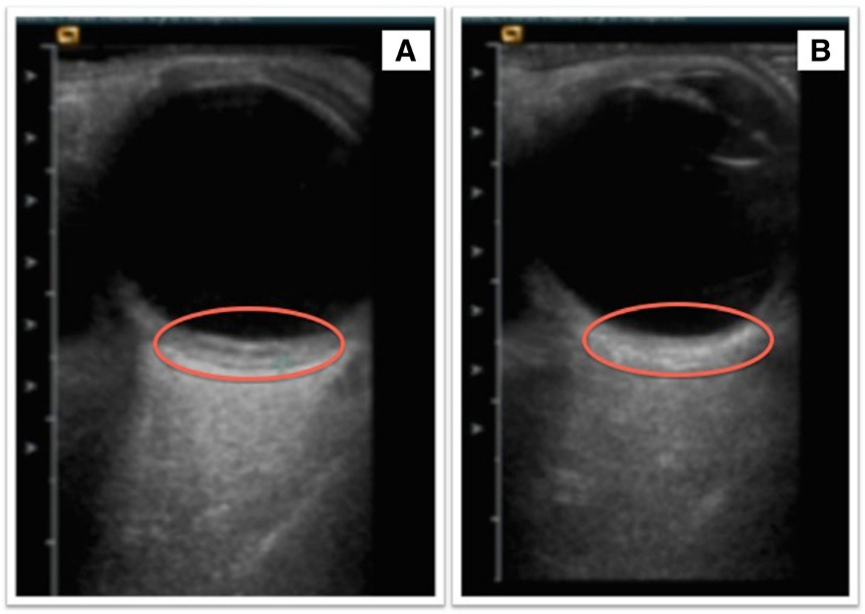
**Ultrasound B-scan images of the right eye.** Pre-treatment showing presence of nodular sessile swelling in the temporal quadrant **(A)** and resolution of the sessile swelling after treatment **(B)**.

On topical and systemic steroids, there was resolution of the hypopyon, and slit-lamp examination showed bilateral corneal oedema with few Descemet's membrane folds (Figure 4A). Follow-up B-scan showed bilateral sessile temporal elevation of 1.7 and 1.9 mm in both eyes (Figure 5A). As the patient was systemically unwell with intractable headache, he was admitted under the care of infectious disease specialists, and neuroimaging with lumbar puncture was performed. No abnormal findings were noted, and cerebrospinal fluid (CSF) analysis did not show evidence of leishmanial DNA on PCR testing or presence of amastigotes. Based on his past history of recurrent VL, including central nervous system involvement, his bilateral chronic granulomatous anterior uveitis with exudative retinal detachment was attributed to presumed ocular leishmaniasis, and he was started on sodium stibogluconate treatment.

Microbiological analysis from vitreous biopsy was unremarkable and negative for microorganisms, including Leishmania, toxoplasmosis, syphilis, tuberculosis, herpes simplex virus (HSV), varicella-zoster virus (VZV) and cytomegalovirus (CMV). As the patient was showing signs of systemic and ocular improvement, he was kept on anti-leishmania therapy plus oral and topical steroids.

On the last follow-up examination in November 2013, the inflammation improved with the visual acuity improving to counting fingers and 6/24 in the right and left eyes, respectively (Figure 4B). Anterior segment examination revealed resolution of inflammation with presence of posterior synechiae and posterior subcapsular cataract in the right eye. He was maintained on sodium stibogluconate and a tapering dose of oral corticosteroids, and his vision continued to improve to 6/9 in the left eye. The ultrasound of the posterior segment showed a complete involution of the sessile nodular granulomatous swelling in both eyes (Figure 5B). Unfortunately, after the last follow-up, the patient expired due to his systemic illness and concurrent infections.

## Discussion

Visceral leishmaniasis (VL) may range from asymptomatic to a disseminated form. Patients with VL and HIV co-infection usually have a CD4 count less than 200 cells/μl, may have involvement of atypical sites, like the eye, and are prone to a relapsing VL course despite appropriate treatment [9],[10]. Leishmaniasis can present with severe fulminant uveitis in patients with highly active anti-retroviral therapy (HAART)-induced immune reconstitution syndrome [11]. HAART has resulted in decrease in incidence of VL especially in the Mediterranean [12]. The recent studies have implicated the inadequacy of the current treatment modality for HIV-associated visceral leishmaniasis [13]. Lifelong prophylaxis is reported to be necessary for HIV-associated visceral leishmaniasis [13].

Ocular leishmaniasis has been described in the literature as chronic blepharo-conjunctivitis and panuveitis [5]-[8]. However, the pathogenesis of this condition is not well established. El Hassan et al. published a short series of six patients with presumed post-kala-azar ocular leishmaniasis with two cases having severe granulomatous panuveitis [5]. None of the cases were confirmed microbiologically. All patients responded to systemic anti-leishmania therapy plus topical steroids. Another series published by Khalil et al. reported five cases of ocular leishmaniasis panuveitis [6]. In this series, two patients lost their sight permanently, but three were successfully treated with oral and topical corticosteroids along with anti-leishmania treatment.

A case of fulminant ocular leishmaniasis in an HIV-positive patient was reported in 2004. This HIV-positive patient on HAART for 2 years was previously diagnosed and treated for VL. He also presented with a severe bilateral granulomatous panuveitis with hypopyon. However, in this case, leishmaniasis was confirmed by positive PCR analysis in the aqueous humour and the cerebrospinal fluid [7].

Another similar HIV-positive case was also reported in 2002. The patient had an initially low CD4 count and lost his only eye despite anti-leishmania therapy. The parasite was not identified until the enucleated eyeball showed severe inflammation containing leishmania amastigotes. This situation was reported as a HAART-induced immune restitution syndrome due to leishmaniasis [8].

Like other cases reported in the literature, our patient with ocular leishmaniasis also has relapsed VL. Though there was no further evidence of leishmanial infection, he responded well to anti-leishmania therapy. The rationale for giving concurrent oral corticosteroid therapy was possible immune reconstitution response as our patient was on HAART therapy. Well known in CMV retinitis, an immune restitution phenomenon has also been described in other conditions. *Mycobacterium avium* subclinical infection has been associated with fever, leucocytosis and lymphadenitis after initiation of HAART [14]. Meningitis has also been reported in patients with latent cryptococcal infection of the central nervous system after starting HAART [15]. Biswas et al. have earlier reported a single case report of macular haemorrhage in both eyes of an immunocompetent patient who was having opportunistic infection with leishmania [16]. In our case, the initial CD4 count was low (117 cells/μl) when the first and mild ocular symptoms were reported. A few months later, subsequent CD4 count increase to 330 cells/μl with concomitant severe granulomatous panuveitis. Systemic corticosteroids were necessary to control the inflammation. Hence, the possibility of immune reconstitution syndrome could not be ruled out in our case, which is quite extensively reported in the literature [17]. Biswas et al. have also reported a case of immune reconstitution in an immune-compromised individual after initiating protease inhibitors [17]. Based on the patient response to treatment, empirical anti-leishmania therapy and systemic corticosteroid may have contributed to prevent ocular morbidity.

## Conclusions

The diagnosis of ocular leishmaniasis-related panuveitis is difficult and requires a high index of suspicion with good knowledge of the past medical history. A bilateral and severe granulomatous uveitis in an HIV-positive patient with previous history of visceral leishmaniasis should make the clinician suspicious of ocular leishmaniasis. Prompt intervention with systemic therapy is necessary to optimize the outcome. A major immune reaction against lingering parasites might play a key role in the pathogenesis of this rapidly sight-threatening condition. Both the infection and the immune reaction should be kept in mind and targeted when treatment is planned.

## Authors’ original submitted files for images

Below are the links to the authors’ original submitted files for images. Authors’ original file for figure 1 Authors’ original file for figure 2 Authors’ original file for figure 3 Authors’ original file for figure 4 Authors’ original file for figure 5

## Abbreviations

cerebral CT: cerebral computed tomography
CMV: cytomegalovirus
CRP: C-reactive protein
CSF: cerebrospinal fluid
HAART: highly active anti-retroviral therapy
HIV: human immunodeficiency virus
HSV: herpes simplex virus
IV: intravenous
MRI: magnetic resonance imaging
OCT: optical coherence tomography
PCR: polymerase chain reaction
VL: visceral leishmaniasis
VZV: varicella-zoster virus
