# Supplementary figures and images for: A case of panuveitis with hypopyon due to presumed ocular leishmaniasis in a HIV patient
Source: J Ophthalmic Inflamm Infect. 2014 Aug 29;4:21. doi: 10.1186/s12348-014-0021-0 (PMC4883982; doi:10.1186/s12348-014-0021-0)

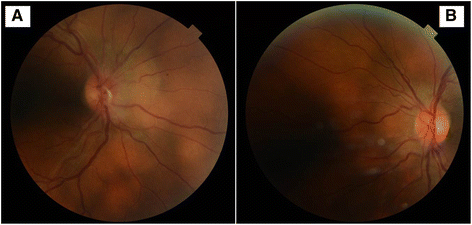

Supplement: Supplementary file 1 — Authors’ original file for figure 1 [file 12348_2014_21_MOESM1_ESM.gif]

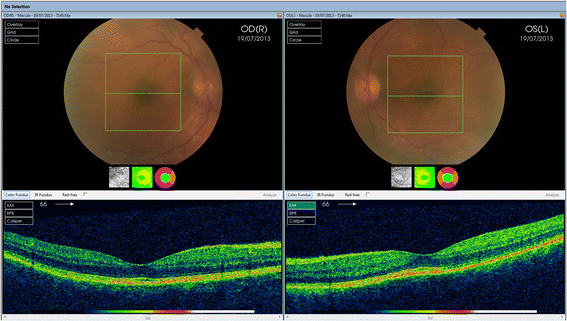

Supplement: Supplementary file 2 — Authors’ original file for figure 2 [file 12348_2014_21_MOESM2_ESM.gif]

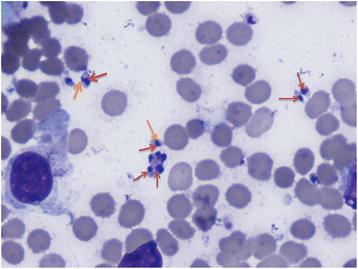

Supplement: Supplementary file 3 — Authors’ original file for figure 3 [file 12348_2014_21_MOESM3_ESM.gif]

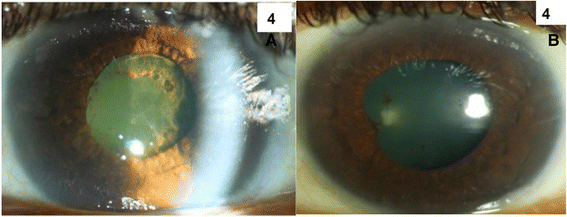

Supplement: Supplementary file 4 — Authors’ original file for figure 4 [file 12348_2014_21_MOESM4_ESM.gif]

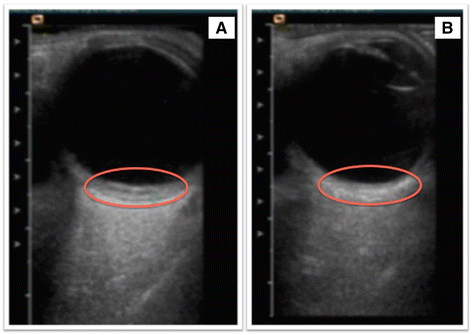

Supplement: Supplementary file 5 — Authors’ original file for figure 5 [file 12348_2014_21_MOESM5_ESM.gif]
